# Cytotoxicity assessment of different doses of ozonated water on dental pulp cells

**DOI:** 10.1186/s12903-021-01392-8

**Published:** 2021-01-19

**Authors:** Ferdiye Küçük, Sibel Yıldırım, Serap Çetiner

**Affiliations:** 1 Department of Pediatric Dentistry, Faculty of Dentistry, Near East University, Nicosia, Mersin 10, Turkey; 2 Department of Pediatric Dentistry, Faculty of Dentistry, Selçuk University, Konya, Turkey; 3 Department of Pediatric Dentistry, Faculty of Dentistry, University of Kyrenia, Kyrenia, Mersin 10, Turkey

**Keywords:** Regenerative endodontics, Ozonated water, Dental pulp cells, Cell proliferation, Irrigation

## Abstract

**Background:**

The purpose of this study was to assess the cytotoxicity of various concentrations of ozonated water (OW) on human primary dental pulp cells.

**Methods:**

Human primary dental pulp cells were isolated from exfoliated primary canine teeth of an 11-year-old patient with good systemic and oral health. Afterwards, cells were divided into 6 experimental groups; four groups of OW in concentrations of 2 mg/L, 4 mg/L, 8 mg/L, and 16 mg/L, untreated control group, and cell culture without cells. Cytotoxicity was evaluated after exposure for 5-min exposure using Mosmann’s Tetrazolium Toxicity (MTT) assay at 0 h and 48 h time points. Data were analyzed using a repeated measures analysis of variance and Post-hoc tests were performed using Bonferroni correction for multiple comparisons.

**Results:**

All experimental groups showed proliferation at 0 h time point. However, all groups also experienced a decrease in overtime at 48 h time point (*p* < 0.05). At both time points 2 mg/L OW showed the highest cell viability as well as proliferation. At 0 h time point, the increase in cell viability for all experimental groups was found statistically significant when compared to positive control group (*p* < 0.05). At 48 h time point, although 8 mg/L and 16 mg/L OW showed statistically significant reduction in compare to 0 h time point, 2 mg/L and 4 mg/L OW groups didn’t experience any statistically significant difference (*p* < 0.05).

**Conclusion:**

Considering our findings, due to ozonated water's induced a higher proliferation rate of dental pulp cells, indicating their biocompatibility and a possible adjuvant on irrigating agent in regenerative endodontic procedures.

## Background

Regenerative endodontics has been defined as “biologically based procedures designed to replace damaged structures, including dentin and root structures, as well as cells of the pulp-dentin complex”[1]. Regenerative endodontic procedure (REP) includes three crucial steps for a successful treatment which are; disinfection of the root canal system, presence of a scaffold, and coronal sealing [2].

Scaffold is essential for regenerative endodontic procedures, it has an adherent role for stem cells and provides proliferation and differentiation of these cells [3].

Bacterial invasion must be avoided after placing of a scaffold or blood clot in the root canal system. In order to do it, a good coronal sealing must be provided for a long-term success of the regenerative endodontic procedures [4].

The success of a regenerative endodontic treatment highly depends on the eradication of microorganisms from root canals [5]. Oral microorganisms form biofilm on root canal space and penetrate into dentinal tubules [6]. Removal of bacteria from root canal system requires efficient disinfection for regenerative endodontic procedures due to deeper penetration of bacteria into dentinal tubules of an immature permanent tooth than a mature tooth [7, 8]. The priority in REP which is a stem cell-based therapy [9] is to maintain root development [10]. It is well-known that vital pulp tissue remnants and apical papilla stem cells (SCAPs) have a crucial role in REP [11]. Therefore, in addition to a proper disinfection of pulp space and dentinal walls, the toxicity level of irrigating solutions to these cells and survival of the cells play an important role in REP [10, 12]. Sodium hypochlorite (NaOCl) is a typically used irrigating agent for regenerative endodontic procedure as recommended by both American Academy of Endodontics (AAE) and European Society of Endodontics (ESE) [13, 14]. NaOCl has a broad spectrum antimicrobial effect and ability to resolve necrotic tissue remnants [15]. Despite its strong antimicrobial efficacy, NaOCl is known as a toxic irrigating agent especially in higher concentrations [16–19]. In addition to its toxicity, there are some concerns about reattachment of degenerated pulp tissue on dentinal walls of root canal [20] and denaturation of dentin matrix proteins which stimulates tissue regeneration [21]. Unless proven otherwise, due to its strong antimicrobial efficacy NaOCl is indispensable for endodontic treatments.

Ozone has a powerful antimicrobial effect against bacteria, fungi and viruses [22] and inhibits the growth of pathogenic organisms such as *Peptostreptococcus micros*,*Enterecoccus faecalis*, *Candica albicans* and *Pseudomonas aeruginosa* [23, 24]. It is reported that it has a high oxidation potential and is 1.5 times stronger than chloride as an antibacterial agent [25]. Ozone can be applied on oral tissues as ozonated water, ozonated oil and in gaseous form. More lately, studies have been focused on antibacterial efficacy of ozonated water as an irrigating agent for conventional endodontic treatments [26–30].

In comparison with 2.5% NaOCl, ozonated water showed almost a similar antimicrobial efficacy and when ozonated water applied on fibroblasts, metabolic activity of cells was increased [24]. However, the antimicrobial efficacy of ozonated water against bacteria embedded in biofilm is suspicious[27]. Ozonated water can be considered as an adjuvant irrigating agent to current regenerative endodontic protocols [24]. Thus, it could be a possible adjuvant irrigating agent in regenerative endodontics. However optimal concentration of ozonated water remained unclear. Besides, biocompatibility of ozonated water has been rarely evaluated in literature [27, 31–33]. Therefore, the purpose of this study was to assess the effects of different concentrations of ozonated water on biological activity of human primary dental pulp cells.

The null hypothesis is;

1. Ozonated water doesn’t proliferate dental pulp cells.
2. Proliferation rate is not concentration and time dependent.

## Methods

### Cell culture

Human primary dental pulp cells were isolated from two healthy primary exfoliated canines of an 11-year-old male patient with good oral and systematic health.

The pulp of the exfoliated deciduous teeth were cut into 1mm^3^ pieces and submitted to enzymatic digestion with 1 mg/ml collagenase type 1 (Sigma Aldrich, Saint Louis, USA) for 1 h at 37 °C with 5% CO_2_. The cells were cultured on 6-well plates including Dulbecco’s modified Eagle’s medium (DMEM) (DMEM High Glucose, Thermo Fischer Scientific, Netherlands) with 15% Fetal Bovine Serum (Life Technologies, USA), 1% Amphotericin-B (Gibco, New York, USA), 1% Penicillin–Streptomycin (Life Technologies, USA), 1% Gentamycin (Life Technologies USA), 1% L-Glutamine (Capricorn Scientific, Ebsdorfengrug, Germany). The cultures were maintained at 37 °C with 5% CO_2_ incubator and the medium was refreshed every 2 days until the cells reached 80% influence. The cells were passed through 3 passages and used for the experiment.

The cells were randomly divided into six experimental groups;

- Group 1 (Positive control): Cells with culture medium
- Group 2: 2 mg/L OW
- Group 3: 4 mg/L OW
- Group 4: 8 mg/L OW
- Group 5: 16 mg/L OW
- Group 6 (Negative control): Distilled water

### Preparation of ozonated water

Ozone Generator (Enaly, OZX-300AT, Shangai, China) was used to prepare ozonated water. The capacity of the ozone generator to produce ozonated water was between a range of 0.1–70 mg/L. The time was assessed to prepare each concentration and ozonation was performed by bubbling ozone through sterile double distilled water according to manufacturer’s instructions. The different concentration: 32 mg/L, 16 mg/L, 8 mg/L and 4 mg/L ozonated water were produced and used freshly before each experiment. The dilution ratio of ozonated water and culture medium was 1:1. Thus, the experimental doses finally were 16 mg/L, 8 mg/L, 4 mg/L, and 2 mg/L.

### Cell viability assay

The cells were seeded on 96-well plates (5 × 10^3^ cells/well) for Mosmann’s Tetrazolium Toxicity (MTT) assay. One well was used for each time interval, with 6 wells per group( 0 h and 48 h).

The cells were treated for 5 min which is considered to be the interaction time of irrigating agents with cells in clinical conditions and cytotoxicity was tested at 0 and 48-h time points. By dissolving 5 mg/ml 2-(3,5-diphenyltetrazol-2-ium-2-yl)-4,5-dimethyl-1,3-thiazole;bromide (Thiazolyl blue tetrazolium bromide, 98%, Acros Organics, China) in Phosphate Buffered Serum(PBS) MTT solution was prepared and filtered. The dilution ratio of this solution with DMEM was 1:9 (1 MTT: 9 DMEM). Culture media was removed and 200 µl of MTT solution were added into each well then incubated at 37 °C for 4 h. Next, MTT solution was replaced by 20 µl of dimethyl sulfoxide (DMSO, Biomatik Corporation, Canada) for each well for solubilizing formazan crystals. The optical density (OD) was read in a microplate reader (Molecular Devices, VersaMax Microplate Reader, USA) at 540 nm. Each condition was analyzed in triplicate.

Percentage of cell viability was calculated according to the formula below;

$$\text{Cell Viability\%}: \frac{\text{OD Value of Experimental Group}-\text{ OD Value of Negative Control Group }}{\text{OD Value of Positive Control Group}-\text{OD Value of Negative Control Group}}* 100$$

### Statistical analysis

In this study, shapes of the distributions of the measured variables were assessed by using Shapiro—Wilk method. The test of normality results indicated that there was normal distrubition of data which suggested that parametric tests could be used for further analyses. Data were detailed with mean ± standard deviation and analyzed using a repeated measures analysis of variance since the data was collected over the two time points, and one of the primary objectives of this study was to observe time-wise variation. Taking steps further with repeated measures, Post-hoc tests were performed using Bonferroni correction for multiple comparisons since between-subject variation does not entail the distinction between the specific groups but overall group-wise difference. Greenhouse-Geeisser correction was considered for the interpretation of the within-level results as the assumption of sphericity had been violated.

The viable cell percentages for ozonated water experimental groups at 0 h and 48 h time points, were detailed and compared with both positive and negative control groups. Statistical analyses were conducted using IBM SPSS software version 25. P values of < 0.05 were considered as significant for the tests results presented here.

## Results

The Group 1 (positive control) and Group 6 (negative control) were considered as 100% and 0%, respectively.

**Fig. 1:**
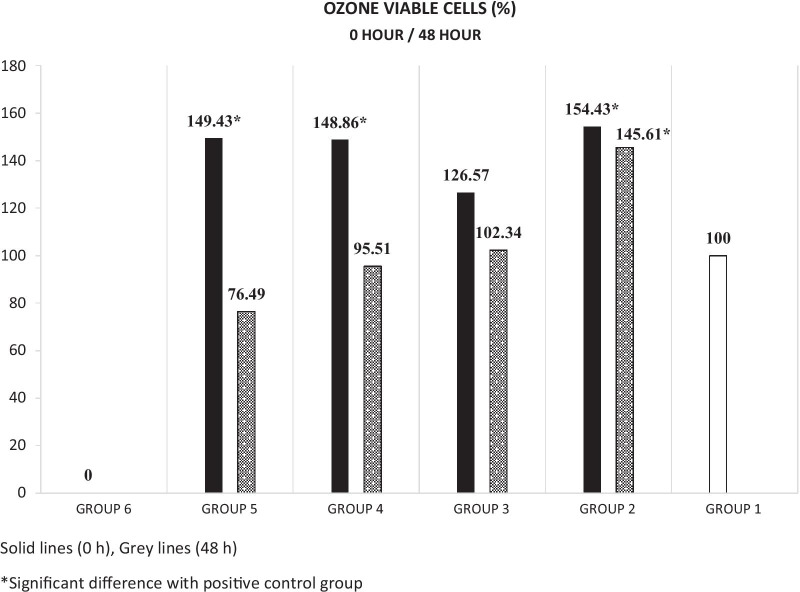
Ozonated water viable cell % over the time and significant difference with positive control group. Solid lines (0 h), Grey lines (48 h)

All experimental groups showed proliferation at 0 h time point. However, all groups also experienced a decrease in overtime at 48 h time point (Fig. 1).

At 0 h time point, 2 mg/L OW showed the highest cell proliferation rate with a mean percentage viable cell value of 154.43 ± 32.13, followed by 16 mg/L OW (149.43 ± 31.10%), 8 mg/L OW (148.86 ± 25.24%) and 4 mg/L OW (126.57 ± 33.28%), respectively (*p* < 0.05) (Table 1). The increase in cell viability in 2 mg/L OW, 4 mg/L OW, and 16 mg/L OW was found statistically significant when compared to positive control group (*p* < 0.05) (Table 2).

**Table 1:** Time-wise comparisons of experimental groups

Group	0 h	48 h	0–48 h	*p*
	Mean ± SD	Mean ± SD	Change (%)	
Group 5	149.43 ± 31.10	76.49 ± 15.26	− 48.81	0.004
Group 4	148.86 ± 25.24	95.51 ± 6.86	− 35.84	0.004
Group 3	126.57 ± 33.28	102.34 ± 28.53	− 19.14	0.151
Group 2	154.43 ± 32.13	145.61 ± 32.11	− 5.71	0.714

*p* values were obtained with Anova test

**Table 2:** Pairwise comparisons of experimental groups

*p* values (0 h/48 h)	Group 6	Group 5	Group 4	Group 3	Group 2
Group 6 (negative control)	–	–	–	–	–
Group 5	0.000/0.000	–	–	–	–
Group 4	0.000/0.000	1.000/1.000	–	–	–
Group 3	0.000/0.000	1.000/0.358	1.000/1.000	–	–
Group 2	0.000/0.000	1.000/0.000	1.000/0.001	0.944/0.006	–
Group 1 (positive control)	0.000/0.000	0.027/0.578	0.030/1.000	1.000/1.000	0.011/0.003

The values presented here are the p values which were obtained with Anova test

The reduction of the mean percentage values of viable cells at 48 h was statistically significant for 8 mg/L OW and 16 mg/L OW when compared to values at 0 h time point (*p* < 0.05). The change between 2 mg/L OW and 4 mg/L OW was not statistically significant when compared to values at 0 h time point and there was still a proliferation effect at 48 h time point in these concentrations. The most uniform result between the two time points was obtained with 2 mg/L OW which reduced from 154.43 ± 32.13% to 145.61 ± 32.11% (Table 1).

At 48 h time point, 2 mg/L OW showed the highest proliferation rate which was statistically significant in comparison to 4 mg/L OW, 8 mg/L OW, 16 mg/L OW, and the positive control group (*p* < *0.05*).

## Discussion

The null hypothesis was rejected. Ozonated water induced proliferation of primary dental pulp cells and proliferation rate was time and concentration- dependent.

The role of infection and inflammation is a challenge for regenerative endodontics [34]. The key to the successful regenerative endodontic therapy is the eradication of microorganisms from the root canal system effectively [6, 35]. NaOCl which has a strong antibacterial efficiency, is used in the current regenerative endodontic protocols of AAE and ESE in low concentrations [13, 14]. Although NaOCl has a considerable antibacterial effect it is also highly toxic on SCAPs. Moreover, previous studies have shown that necrotic immature teeth may contain vital pulp cells as well which also could be affected by the toxicity of irrigating agents [36, 37]. Regarding the side-effects of NaOCl, the wide open apex and/or resorbed apex has been described as a condition where ozone can be an adjuvant irrigant to NaOCl [38].

In a previous study, effects of both gaseous ozone and ozonated water on human oral epithelial cells have been discussed. The results of the study have revealed that ozonated water is more biocompatible than gaseous ozone [33]. For this reason, ozonated water was involved instead of gaseous ozone in the present study.

In recent years, more studies have been made on the antimicrobial efficiency of ozonated water. Previous studies have proven the antimicrobial efficacy of OW, however, authors have failed to agree on an exact dose to be used more sufficiently as an irrigating agent [26–29, 31, 39, 40]. Nagayoshi et al. [27] concluded that the antimicrobial efficacy of ozonated water between the concentrations of 0.5–4 mg/L was highly effective and rapid on killing both gram-positive and gram-negative bacteria. Nogales et al. [31] have considered antimicrobial activity of ozonated water in concentrations of 2, 5 and 8 mg/L. Cardoso et al. [28], evaluated the effect of 24 mg/L OW against E. faecalis and endotoxins in root canals. Considering the proven doses that have antibacterial efficiency in these studies, 2 mg/L, 4 mg/L, 8 mg/L, and 16 mg/L OW have been used in the present study.

Although the antibacterial effect of ozone therapy has proven, cytotoxic or/and proliferative effect of ozonated water is rarely discussed [26, 31–33]. Therefore, the present study aimed to assess the biological response of dental primary pulp cells to various doses of ozonated water by using MTT assay.

MTT assay is one of the most commonly used colorimetric assays to evaluate cytotoxicity [41] and it simply measures cytotoxicity based on the mitochondrial activity of cells. It is easy to use, has a high reproducibility, and it is widely used to determine both cell viability and cytotoxicity [42].

An adequate cell line selection is an important issue for cytotoxicity assessment. Permanent cell lines, standard cell lines and primary cells collected from gingiva, periodontium or pulp are the recommended alternatives. Primary cell line were used in this study which has better ability to represent clinical conditions[43].

The results of the present study revealed that ozonated water was biocompatible in each concentration, and also the proliferation rate of dental primary pulp cells was induced. Ozonated water has distinctive properties such as high oxidizing power and increased intracellular metabolic activity. Thus, high proliferation rate of dental primary pulp cells can be associated with these properties. Although the mean percentage value of viable cells decreased for 16 mg/L OW and 8 mg/L OW at 48 h time point, both groups cannot be considered as toxic because the viable cell percentages were above 70 [44].

Nagayoshi et al. [26] observed that 4 mg/L OW is biocompatible although there was not a statistically significant difference when compared to mean OD values of distilled water. Our results are in agreement with the mentioned study but yet there are differences in methodology. While the authors examined only one concentration of ozonated water, present study includes a wide range of ozonated water in four different concentrations. We have standardized the results by converting the OD values into viable cell percentages, however, Nagayoshi et al. [26] declared results in mean OD values. For this reason, we are not able to compare our cell viability results in percentages. In addition, contact time of the cells with irrigating agents was set as 2 min in the previous study but in the current study the contact time was set as 5 min and the MTT was applied in two separate time points. Required irrigation time for regenerative endodontic protocol for NaOCl was taken in consideration while determining 5 min as the contact time with cells and irrigating agents in our study [13]. Nagayoshi et al. [26] did not mention any induced proliferation by ozonated water at contact time but our results showed that there is a highly induced proliferation in every concentration of ozonated water at 0 h time point which is statistically significant when compared to positive control group except Group 3 (*p* < *0.05*). In another point of view, induced proliferation in the experimental groups may be associated with a high proliferation rate of dental primary pulp cells.

The experimental design of Nogales et al. [31] was almost similar to the present study. The authors included 2 mg/L, 5 mg/L, and 8 mg/L OW concentrations in this study. According to this study, there is an increase in mean percentage values of viable cells over time and proliferation did not occur at 0 h time point of all concentrations of ozonated water. Moreover, at 48 h time point, Nogales et al. [31] pointed that the highest proliferation rate was achieved by the 8 mg/l OW which is the highest concentration used in the study and the only cytotoxic concentration at 0 h time point with the mean percentage value of 68.6.

In the present study, 2 mg/L OW gave the most stable results between two time points, which means that there was not a significant change in cell viability over time. When we compare our result with Nogales et al. [31], it can be observed that the 2 mg/L OW is the most stable ozonated water concentration among all time points as well, no dramatically high increase was observed. However, cell viability percentage of 2 mg/L OW in our study is higher than the previous study. This can be attributed to higher proliferation rate of our dental primary pulp cells.

In another study, cytotoxicity of OW with concentrations of 5 mg/L, 10 mg/L, and 20 mg/L were tested by using MTT assay at 5, 10 and 15-min time points of interaction with stem cells from human exfoliated deciduous teeth (hSHEDs). The study revealed that the 20 mg/L OW at 5 and 10-min time points showed the highest proliferation rate whereas the 5 mg/L ozonated water showed the highest proliferation rate at the 15-min time point [32]. The authors evaluated the cytotoxicity by using MTT assay and the cell viability results were given in percentages. Although hSHEDs which have high capacity of proliferation were preferred in their study, the proliferation rate was not as high as the proliferation rate in our results. This may be due to the cells’ going through a cryopreservation procedure before the experiment or to the contact time which differs from our methodology.

Considering the results of this study 2 mg/L OW is recommendable as a possible adjuvant irrigating agent for the regenerative endodontic procedures. Due to the biocompatibility of OW, in addition to a sufficient disinfection, it provides an environment which supports the tissue engineering strategies and achievement of a success in pulp repair or pulp regeneration. However, further studies are needed to evaluate the biological response and odontoblastic differentiation mechanism of SCAPs to ozonated water. Future studies should also focus on interactions between ozonated water and common irrigating solutions such as NaOCl, ethylenediamine tetraacetic acid and citric acid. In addition, for the evaluation of recovery ability of the cells, a long-term experimental period can be planned, different cytotoxicity evaluation methods can be preferred and number of the patients can be increased to have a cell proof for further studies.

## Conclusion

Within the limits of this study, it could be concluded that the ozonated water is non-toxic and induces proliferation of cells as well. This proliferation effect was time and dose-dependent.

## Data Availability

The datasets generated or analyzed during the current stud are available from the corresponding author upon reasonable request.

## Abbreviations

MTT: Mosmann’s Tetrazolium Toxicity
REP: Regenerative endodontic procedures
SCAPs: Apical papilla stem cells
NaOCl: Sodium hypochlorite
AAE: American Academy of Endodontics
ESE: European Society of Endodontics
OW: Ozonated water
DMEM: Dulbecco’s modified Eagle’s medium
OD: Optical density
